# Supplementary material for: Topical Administration of Lactiplantibacillus plantarum (SkinDuoTM) Serum Improves Anti-Acne Properties
Source: Microorganisms. 2023 Feb 7;11(2):417. doi: 10.3390/microorganisms11020417 (PMC9967017; doi:10.3390/microorganisms11020417)
Supplement: Supplementary file 1 [file microorganisms-11-00417-s001.zip › microorganisms-2197314-supplementary.pdf]

Supplementary Materials:

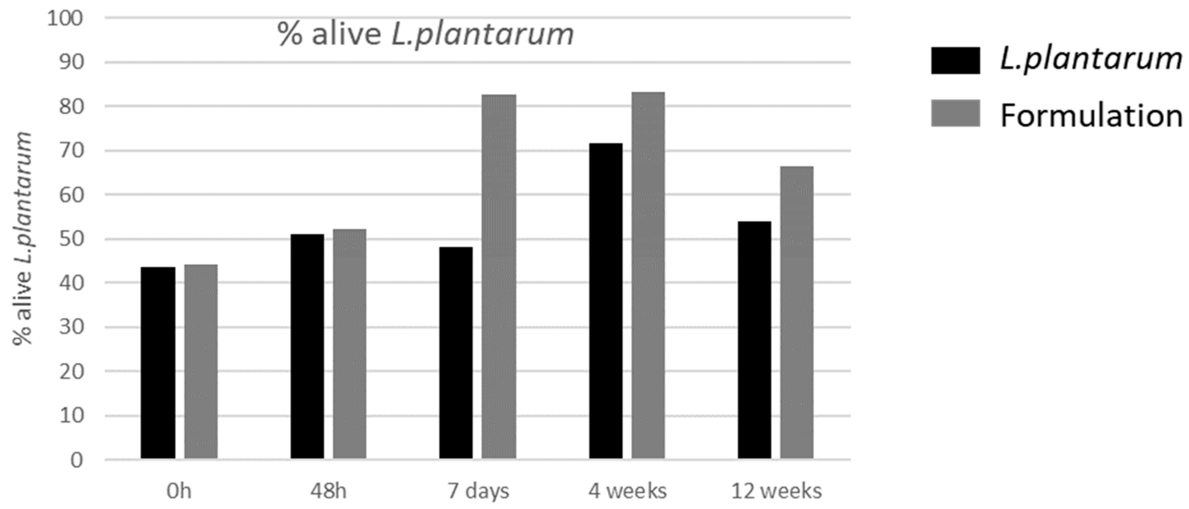

Figure S1. Percentage of Alive *L. plantarum* compared to complete formulation over time. The serum formulation SkinDuo™ was reconstituted with sterile water, after time points: 0 h, 48 h, 7 days, 4 weeks and 12 weeks. For each condition the ratio of the average CFU/reaction value for PMA-qPCR samples over the average CFU/reaction value for Total live bacteria was calculated as ratio of live bacteria for each condition tested.

Table S1. Skin irritation patch tested on 25 healthy subjects.

Sample	Sex	Erythema 15′	Oedema 15′	Erythema 1h	Oedema 1h	Erythema 24h	Oedema 24h
1	m	0	0	0	0	0	0
2	f	0	0	1	0	1	0
3	m	0	0	0	0	0	0
4	f	0	0	0	0	0	0
5	f	0	0	0	0	0	0
6	f	0	0	0	0	0	0
7	m	0	0	0	0	0	0
8	f	0	0	0	0	0	0
9	f	0	0	0	0	0	0
10	f	0	0	0	0	0	0
11	f	0	0	0	0	0	0
12	m	0	0	0	0	0	0
13	m	0	0	0	0	0	0
14	m	0	0	0	0	0	0
15	f	0	0	1	0	0	0
16	f	0	0	0	0	0	0
17	m	0	0	0	0	0	0
18	m	0	0	0	0	0	0
19	m	0	0	0	0	0	0
20	f	0	0	0	0	0	0
21	f	0	0	0	0	0	0
22	f	0	0	0	0	0	0
23	m	0	0	0	0	0	0
24	m	0	0	0	0	0	0
25	f	0	0	0	0	0	0

Table S2. *L. plantarum* viability as CFU/mL on hyposkin® models after SkinDuo™ treatment for two independent experiments in triplicates, showing the mean and SD in CFU/mL. One vial was used and tested for SkinDuo™ n=1, n=2 tested at times 0 h, 8 h, 24 h and 48 h.

**Serum Skin Duo™**	**n=1**			**n=2**			**Mean/CFU/mL**	**SD**
Time	5.56×10^{6}	6.12×10^{6}	5.64×10^{6}	5.56×10^{6}	6.12×10^{6}	5.64×10^{6}	5.77×10^{6}	247296.5
0 h	3.54×10^{6}	3.70×10^{6}	4.06×10^{6}	5.22×10^{6}	4.60×10^{6}	4.82×10^{6}	4.32×10^{6}	605355.7
8 h	2.00×10^{6}	3.20×10^{6}	3.50×10^{6}	2.60×10^{5}	3.62×10^{6}	2.00×10^{6}	2.43×10^{6}	1171367
24 h	3.36×10^{6}	4.32×10^{6}	3.50×10^{6}	1.64×10^{7}	1.83×10^{7}	1.42×10^{7}	1.00×10^{7}	6415278
48 h	1.64×10^{7}	1.74×10^{7}	1.91×10^{7}	2.15×10^{7}	1.28×10^{7}	1.37×10^{7}	1.68×10^{7}	2991174
